# Eptifibatide-induced acute profound thrombocytopenia: a case report

**DOI:** 10.1186/1756-0500-7-107

**Published:** 2014-02-25

**Authors:** Christos Graidis, Christos Golias, Dimokritos Dimitriadis, Georgios Dimitriadis, Theodosis Bitsis, Ilias Dimitrelos, Afroditi Tsiakou, Konstantinos Charalabopoulos

**Affiliations:** 1 Department of Physiology, Clinical Unit, Medical Faculty, Democritus University of Thrace, Alexandroupolis 68100, GR, Greece; 2 Department of Cardiology, Serres State Hospital, Serres, GR, Greece; 3 Department of Interventional Cardiology, Kyanous Stavros, Thessaloniki, Greece

**Keywords:** Eptifibatide, Thrombocytopenia, IIb/IIIa receptor antagonists, Cell adhesion molecules

## Abstract

**Background:**

The interactions among cells or among cells and components of the extracellular matrix, is a crucial pathophysiological process involving some molecules collectively known as adhesion molecules (CAMs). Glycoprotein IIb / IIIa receptors are only restricted to blood platelets and they bind fibrinogen and adhesion proteins such as fibronectin, vitronectin, von Willebrand factor to form cross bridges between adjacent platelets. IIb/IIIa receptor antagonists are an object of intense research activity for target therapy worldwide during the last decades. Three GPIIb/IIIa inhibitors, abciximab, tirofiban, and eptifibatide, have been approved for clinical use. Profound thrombocytopenia is an uncommon but clinically important complication of glycoprotein IIb/IIIa inhibitors.

**Case presentation:**

This case report discusses a forty-four-year-old male patient with acute coronary syndrome who underwent percutaneous coronary intervention and developed profound thrombocytopenia within 4 hours of first administration of eptifibatide.

**Conclusion:**

This report adds another case of eptifibatide-induced thrombocytopenia to the medical literature and endorses the importance of platelet count monitoring after initiating therapy with this agent.

## Background

The interactions among cells or among cells and components of the extracellular matrix, is a crucial pathophysiological process involving molecules collectively known as adhesion molecules (CAMs). CAMs are ubiquitously expressed proteins with a key function in physiological maintenance of tissue integrity and an eminent role in various pathological processes such as cardiovascular disorders, atherogenesis, atherosclerotic plaque progression and regulation of the inflammatory response. CAMs such as selectins, integrins, and immunoglobulin superfamily take part in interactions between leukocyte and vascular endothelium (leukocyte rolling, arrest, firm adhesion, migration). Integrins are a family of adhesion molecules performing a major role in such multiple cellular functions including carcinogenesis and metastatic process. The GP IIb/IIIa receptors (fibrinogen or aggregation receptors), belong to the family of integrins which are membrane bound adhesion molecules and are made of two glycoprotein sub-units (a and b). GP IIb/IIIa receptors are only restricted to blood platelets and they bind fibrinogen and adhesion proteins such as fibronectin, vitronectin and von Willebrand factor to form cross bridges between adjacent platelets. IIb/IIIa receptor antagonists are an object of intense research activity for target therapy worldwide during the last decades and they are frequently used during percutaneous coronary intervention (angioplasty with or without intracoronary stent placement) as well as treating acute coronary syndromes, without percutaneous coronary intervention. Three GPIIb/IIIa inhibitors, abciximab, tirofiban, and eptifibatide, have been approved for clinical use. All are given by intravenous administration, usually for 12 to 18 h after the patient undergoes angioplasty [1-4]. This case report discusses forty-four-year-old male patient who developed profound thrombocytopenia within 4 hours of first administration of eptifibatide.

## Case presentation

**Figure 1:**
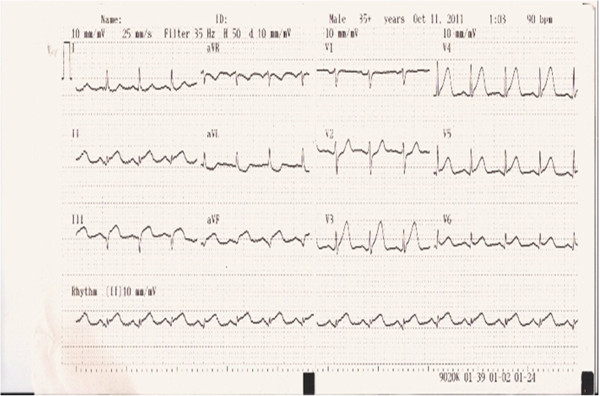
Patient’s electrocardiogram on admission.

**Figure 2:**
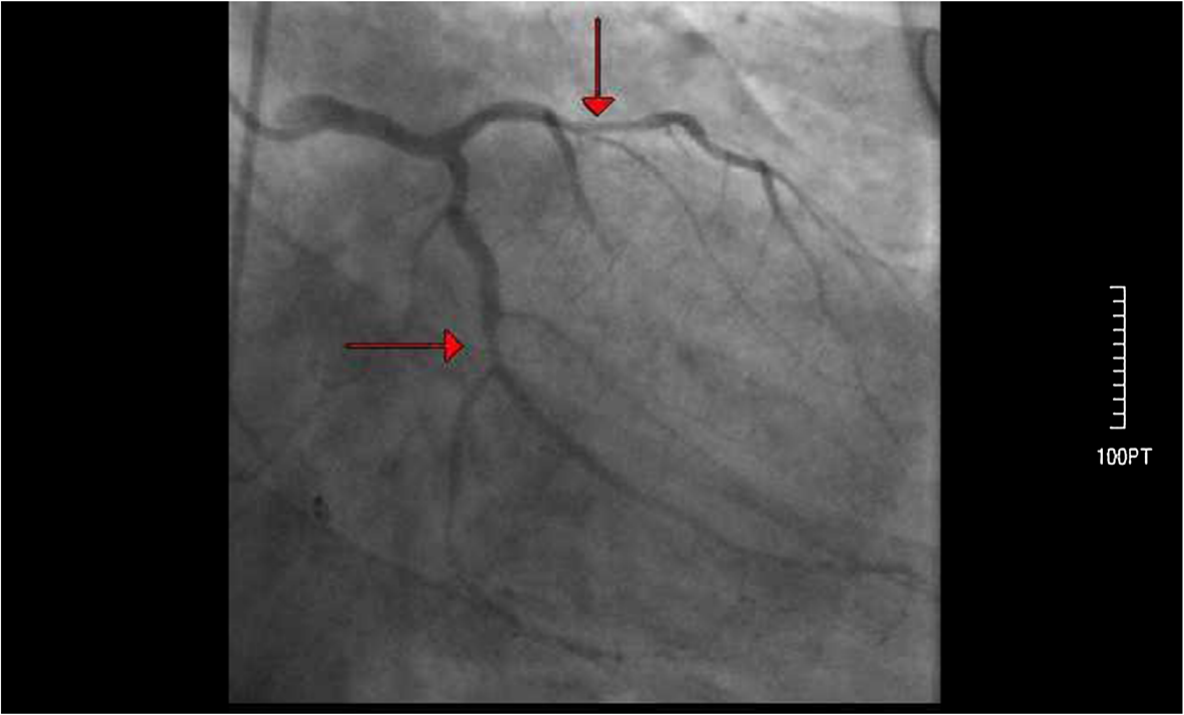
**Coronary angiography of the left coronary artery after admission to the hospital.** Arrows indicate longitudinal critical stenosis of 70% immediately after the origin of a large diagonal branch and a 70% stenosis at the level of the bifurcation with the first obtuse marginal branch of the left circumflex artery.

**Figure 3:**
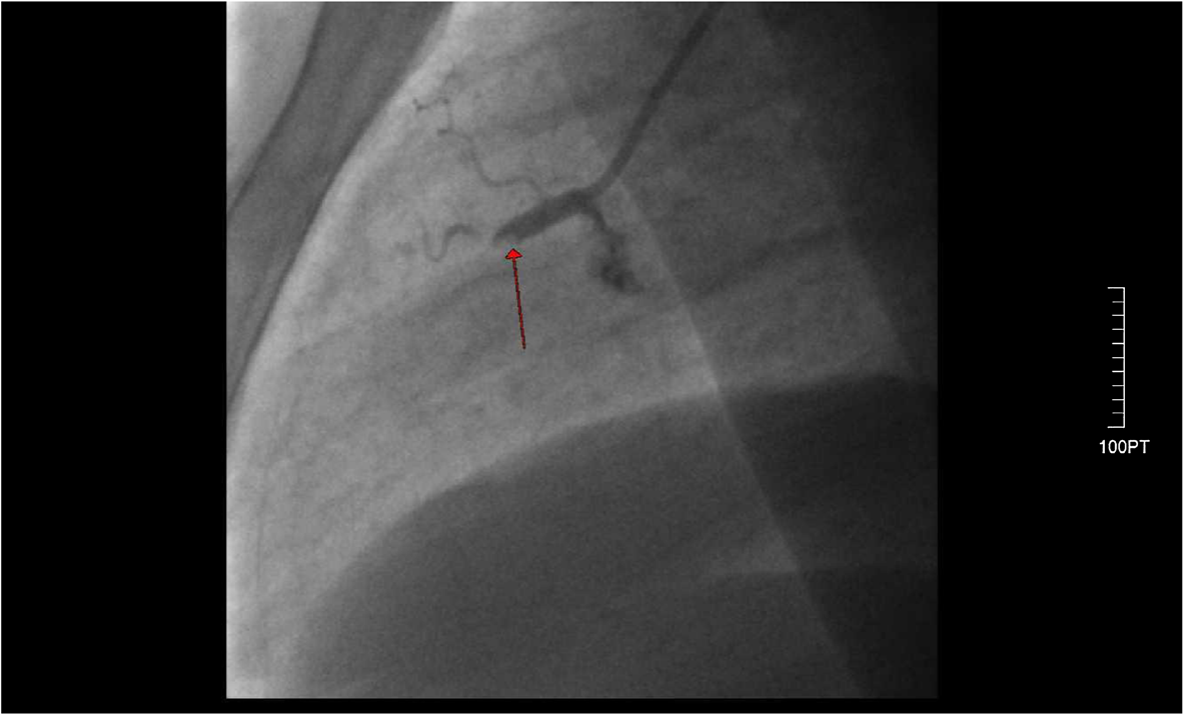
The right coronary artery was totally occluded (red arrow pointing to the lesion) with a residual thrombus.

A 44-year-old Caucasian male with no previous history of cardiovascular disease presented to the emergency department of the hospital with a two-hour history of retrosternal chest pain radiating to the left arm and mandible. He denied any previous history of blood dyscrasia or thrombocytopenia. He had no history of cardiac disease, drug abuse, and he mentioned two cardiovascular risk factors (tobacco abuse and hyperlipidemia). Additionally, he denied any history of a previous hospitalization where he may have received heparin or eptifibatide. His electrocardiogram (ECG) showed sinus rhythm with diffuse ST elevation of the II, III, aVF, V3 to V6 leads and reciprocal changes in I, aVL (Figure 1) without any hemodynamic compromise (blood pressure 120/85 mmHg). His early management included treatment with intravenous unfractionated heparin (5000 unit bolus) followed by an infusion of 18 units/kg/hr, aspirin 325 mg, clopidogrel 600 mg, iv nitrates at a constant infusion, b-blockers (metoprolol 50 mg), intravenous morphine (4 mg), and oxygen 2 l/min. The patient had a white blood cell count of 11.000/mm^3^, a hemoglobin level of 14.0 g/dL, and a platelet count of 220,000/mm^3^. Values of prothrombin time (PT) and activated partial thromboplastin time (aPTT) were within normal limits. Due to the fact that the hospital was unable to perform percutaneous coronary intervention (PCI) or transfer the patient to a tertiary institute at a time less than 120 minute to PCI (door to needle), fibrinolytic therapy was decided upon and performed (tenecteplase 50 mg iv bolus) in the absence of any contraindications (absolute or relative). The symptoms 60 min after the medical revascularization did not reside, the ST-elevation remained unchanged, and reperfusion arrhythmias were not noticed. Taking into consideration all the above reasons, the patient was immediately transferred to the cardiac catheterization laboratory of our clinic for a rescue PCI. Coronary angiography showed that the left main coronary artery (LMCA) was a wide atheromatic vessel without critical stenoses, the left anterior descending coronary (LAD) artery was a relatively large vessel, with sparse atheromatic plaques and revealed a longitudinal critical stenosis of 70% immediately after the origin of a large diagonal branch (Figure 2). The left circumflex artery (LCx) had a 70% stenosis at the level of the bifurcation with the first obtuse marginal branch (Figure 2). The right coronary artery was totally occluded with a residual thrombus (Figure 3), with a Thrombolysis in Myocardial Infarction (TIMI) flow of 0. Protherapy (class IIa indication) with eptifibatide was decided due to the increased thrombus load of the right coronary artery (RCA) for at least 1 hour as an adjuvant measure before proceeding to the percutaneous transluminal coronary angioplasty (PTCA). The patient received an intravenous double bolus of 180 μg/kg of eptifibatide (10 minutes apart), followed by a 2 μg/kg/min infusion.

**Figure 4:**
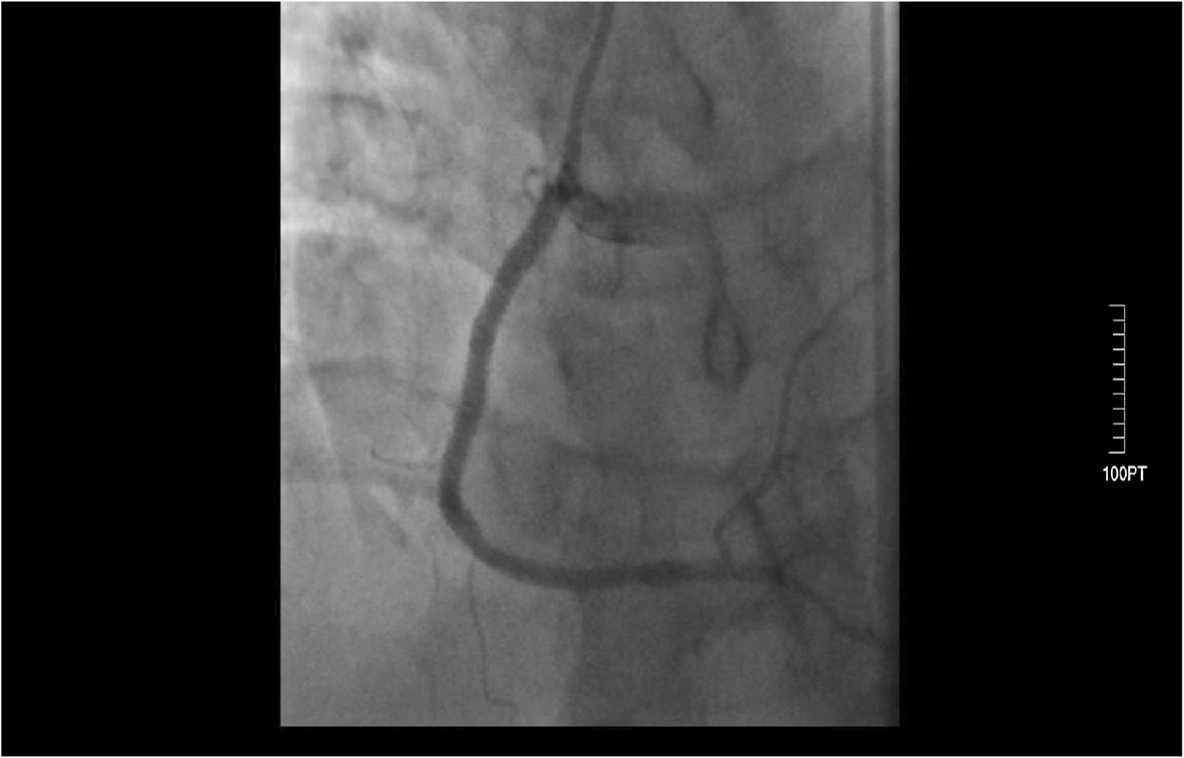
Successful stenting of the right coronary artery.

A 3.0×24 mm PROMUS™ Element™ stent was deployed in the LAD with a very satisfying angiographic result (TIMI 3), the lesion of the LCx was also managed with the use of 3.0×24 mm PROMUS™ Element™ stent achieving an excellent result (TIMI 3). At the right coronary artery, thrombus extraction catheter (Thrombuster II) was used to remove the discrete, intraluminal filling defect that was noted within the infarct-related artery. Multiple passages were carried out in order to restore the flow and an important amount of thrombus load was aspirated using a 6-Fr Thrombuster II. The patient then underwent successful stenting of the right coronary artery (Figure 4) with the deployment of a 3.0×32 mm PROMUS™ Element™ distally and 3.0×20 mm PROMUS™ Element™ proximally. Once flow was restored in the RCA, the patient became pain-free and had resolution of ST segment elevation. He was transferred in a stable condition to the coronary care unit. Post-percutaneous coronary intervention medications included aspirin 100 mg po daily, ramipril 5 mg po daily, metoprolol succinate 50 mg per os daily, clopidogrel 75 mg po daily, rosuvastastin 20 mg po daily, and the eptifibatide infusion was to be continued for 18 hours.

**Figure 5:**
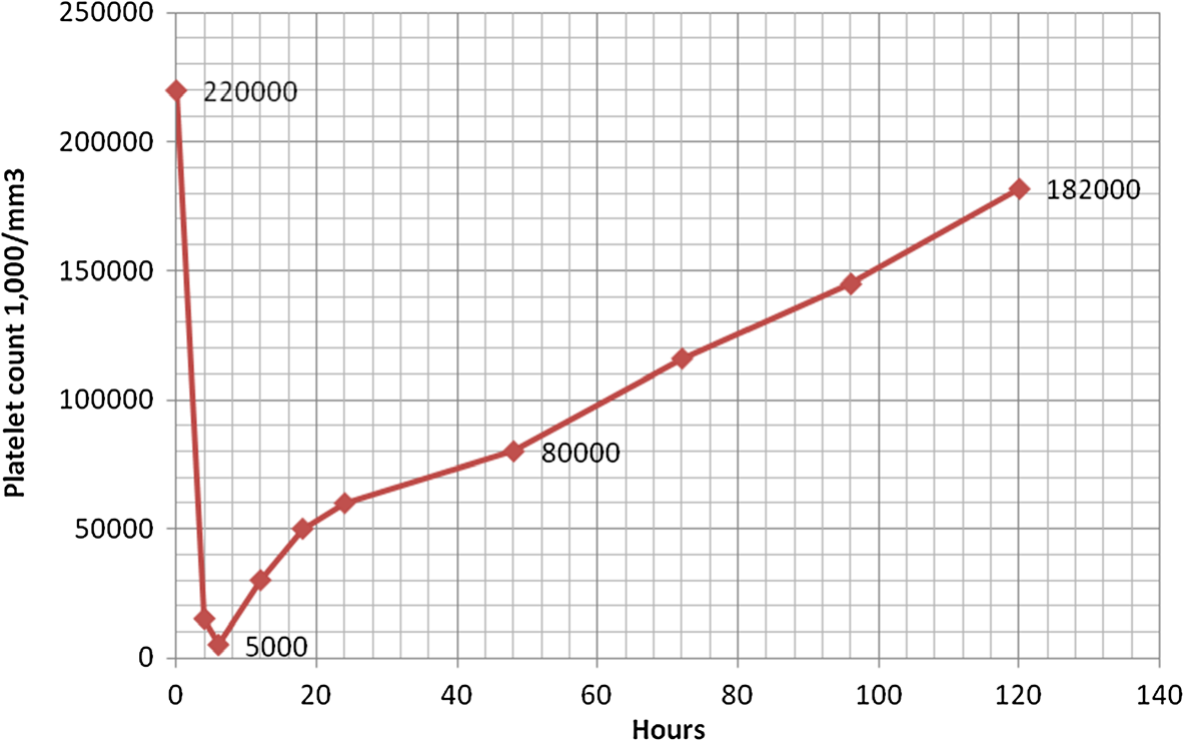
**Chart presenting the development of profound thrombocytopenia, with a platelet count dropping by over 90% from baseline to 15,000/mm**^**3 **^**during the first hours.**

Approximately four hours post-percutaneous coronary intervention and eptifibatide initiation, the patient developed profound thrombocytopenia, with his platelet count dropping by over 90% from baseline to 15,000/mm^3^ while the hemoglobin level remained stable (Figure 5). A peripheral blood smear showed no signs of platelet clumping, ruling out pseudothrombocytopenia and no evidence of microangiopathic hemolytic anemia. All antiplatelet products including heparin, eptifibatide as well as dual antiplatelet therapy (DAPT) was discontinued for 48 hours due to the profound thrombocytopenia 15,000/mm^3^, outweighing the risk of an early stent thrombosis with the risk of a fatal bleeding event such as an intercranial hemorrhage that would suspend the dual antiplatelet therapy for an indefinitive time period. Moreover patient related factors for acute stent thrombosis (<24 h of implantation) such as renal failure, diabetes mellitus and low ejection fraction were not present to our patient [5]. The patient was thereafter consulted to our hematology department. The patient’s platelet level reached its nadir (5,000/mm^3^) approximately 6 hours post-eptifibatide initiation. A heparin-induced thrombocytopenia (HIT) [6,7] platelet factor 4 antibody test was negative [8,9]. The patient was closely observed for any bleeding events. Platelets (5 packs, total) were transfused in the next 24 hours. After the transfusions, the platelet count increased from 5,000/mm^3^ to 60,000/mm^3^. Subsequently, the patient’s platelet count continued to rise (80,000/mm^3^) 48 hours after the eptifibatide exposure, which enabled us to restart both aspirin and clopidogrel. The patient showed no signs of active bleeding, bruises, ecchymosis, or petechiae during the hospitalization and was discharged on day five having a platelet count of 182,000/mm^3^, free of symptoms.

## Conclusions

While eptifibatide presents significantly improved outcomes in patients undergoing percutaneous coronary intervention and among those presenting with an acute coronary syndrome, a small number of patients given eptifibatide develop acute profound thrombocytopenia (20.000 cells/mm^3^) within hours of receiving the drug that can increase the risk of serious bleeding with sometimes deleterious effects. The disorder appears to occur less frequently in clinical trials of eptifibatide, the heptapeptide small molecule GPIIb-IIIa inhibitor. In the PURSUIT trial (Platelet Glycoprotein IIb/IIIa in Unstable Angina: Receptor Suppression Using Integrilin Therapy), which involved patients with non-ST-elevation acute coronary syndrome, acute profound thrombocytopenia occurred in five (0.1%) of 4614 patients treated with eptifibatide and in two of 4603 patients who received placebo [10]. In the ESPRIT trial (Enhanced Suppression of the Platelet IIb/IIIa Receptor with Integrilin Therapy) of non emergent coronary angioplasty with stenting, acute profound thrombocytopenia occurred in two (0.2%) of 1040 patients treated with eptifibatide and in none of the 1024 placebo-treated patients [11]. In the Integrilin to Minimize Platelet Aggregation and Coronary Thrombosis (IMPACT-II) the incidence of thrombocytopenia approximates the 2.8% [12]. Profound thrombocytopenia is an uncommon but clinically important complication of glycoprotein IIb/IIIa inhibitors [13-15].

Drug induced profound thrombocytopenia [16], a predictor of adverse outcome in patients with acute coronary syndromes, is defined as a decrease in platelet count to below 20,000/mm^3^ within 24 hours of exposure to the drug and may be associated with the administration of eptifibatide, a glycoprotein antagonist. While the usage of GP IIb/IIIa inhibitors sets an important clinical benefit in the management of ACS, the reporting incidence of acute profound thrombocytopenia induced by eptifibatide is 0.1% to 1% and usually occurs within the first 24 hours. Several pathophysiological mechanisms have been implicated in the explanation of GP IIb/IIIa inhibitors induced acute profound thrombocytopenia. Accumulating evidence has indicated that pre-existing drug dependent antibodies to platelet surfaces are present in patients and that the glycoprotein inhibitors may induce a change in the conformation in the GP IIb/IIIa receptors on the platelet surface, leading to the expression of neoepitopes which are recognized by pre-existing serum antibodies [17,18]. Another possible mechanism would be the formation of a receptor-antagonist complex by the recognized neoepitopes. In our patient, the physiological platelet count and all other hematological parameters (PT, aPTT, red blood cells, white blood cells) ruled out bone marrow dysfunction, non-immune and immune thrombocytopenia as a cause of thrombocytopenia. Pseudothrombocytopenia was ruled out by manual examination of blood film [19]. There is no evidence in the literature which suggests that the use of tenecteplase is responsible for provoking thrombocytopenia. Not even in the drug monography summary of product characteristics (spc) is mentioned the likelihood of emerging thrombocytopenia after the use of this drug. Additionally, serotonin release assay (HIT panel) came back negative. The fact that the patient continued to be on clopidogrel without recurrence of thrombocytopenia after the normalization of platelet count ruled out clopidogrel as a cause of thrombocytopenia. No history of prior eptifibatide exposure was present and the Naranjo scale score [20] was 5, indicating a possible relationship between the adverse effect and therapy in this patient. There is an algorithm that has been proposed for the early evaluation and management of this drug related thrombocytopenia. In all patients that receive GP IIb/IIIa inhibitors a complete blood count should be obtained prior to treatment, within 2 hours following the intravenous bolus, in a daily basis after the intervention and again prior to the patient’s discharge [21,22].

This report adds another case of eptifibatide induced thrombocytopenia to the medical literature and endorses the importance of platelet count monitoring after initiating therapy with this agent.

## Consent

Written informed consent was obtained from the patient for publication of this Case Report and any accompanying images. A copy of the written consent is available for review by the Editor-in-Chief of this journal.

## Competing interests

The authors declare that they have no competing interests.

## Authors’ contributions

GrCh had a substantial contribution in drafting the manuscript, performing, analysis and interpretation of the coronary angiography data; GCh made substantial contributions to the conception, design, drafting, and critical revision of the manuscript. He gave final approval for publication. DG examined the patient, analysed and interpreted the data regarding hospital admittance. He had substantial contribution to conception, in drafting the manuscript and revising it critically along with analysis and interpretation of echocardiographical data; DD had substantial contribution in drafting the manuscript, performing, analysis and interpretation of coronary angiography data; BTh and DI showed substantial contribution in collection and acquisition of data, drafting the manuscript. AT had contribution in drafting and revising the manuscript; CK made substantial contributions to conception and design, acquisition of data, analysis and interpretation of data. He was involved in revising the manuscript critically and gave final approval for publication. All authors read and approved the final manuscript.
